# Supplementary figures and images for: Learning context shapes bimanual control strategy and generalization of novel dynamics
Source: PLoS Comput Biol. 2023 Dec 8;19(12):e1011189. doi: 10.1371/journal.pcbi.1011189 (PMC10732368; doi:10.1371/journal.pcbi.1011189)

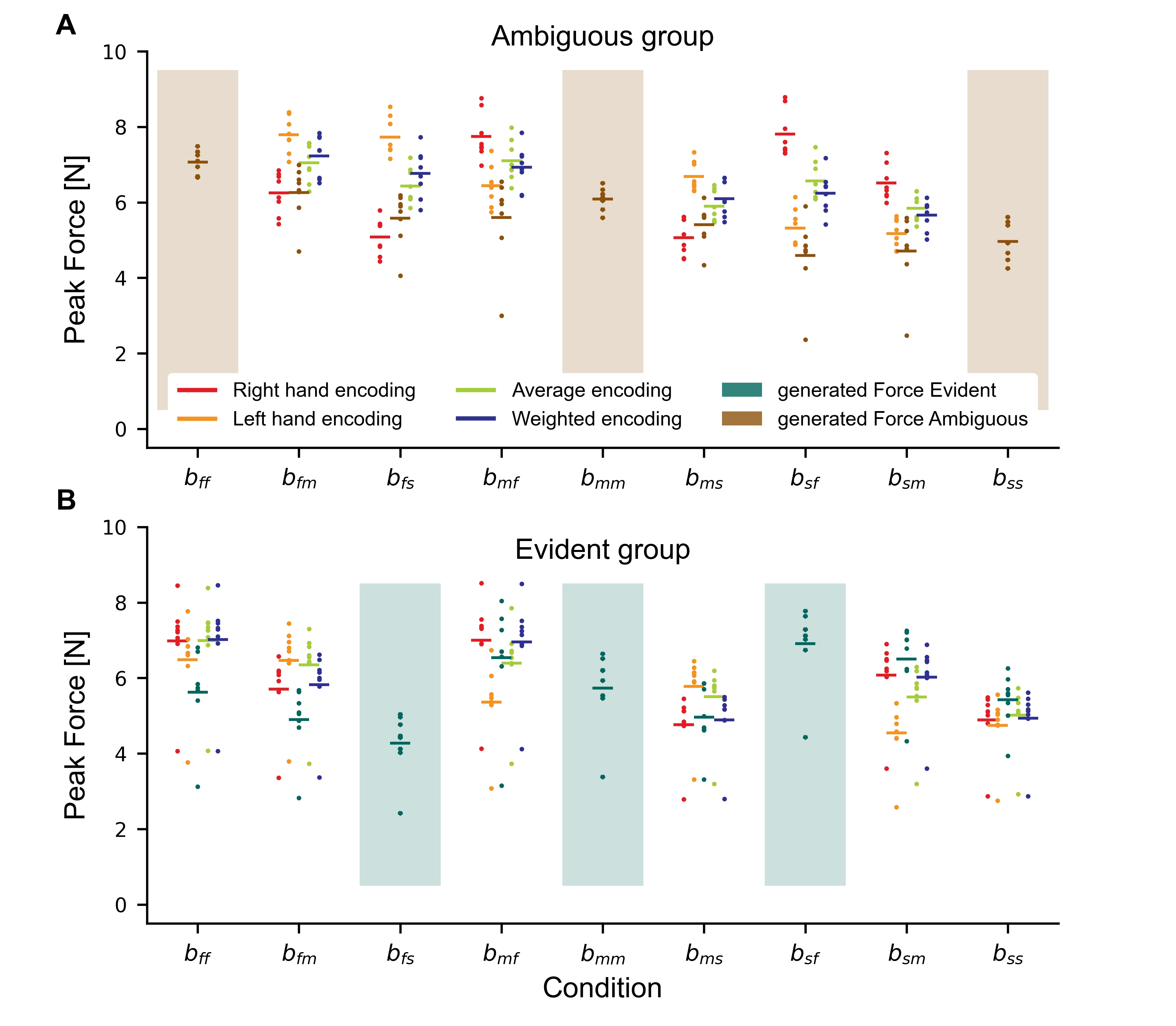

Supplement: S1 Fig — The horizontal lines depict the group average and dots individual participants. The right-hand encoding shows the required force induced by the force field respective the individual adaptation for each participant. (A), The ambiguous group generated peak forces, which deviated from the required forces (red), especially in the conditions bmf, bsf, and bsm. (B), The evident group scaled the peak forces inversely during the trained conditions, and were able to generalize to the generalized conditions (as indicated by their adherence to the right-hand encoding). However, generalization was less strong for bff and bfm. (TIF) [file pcbi.1011189.s001.tif]

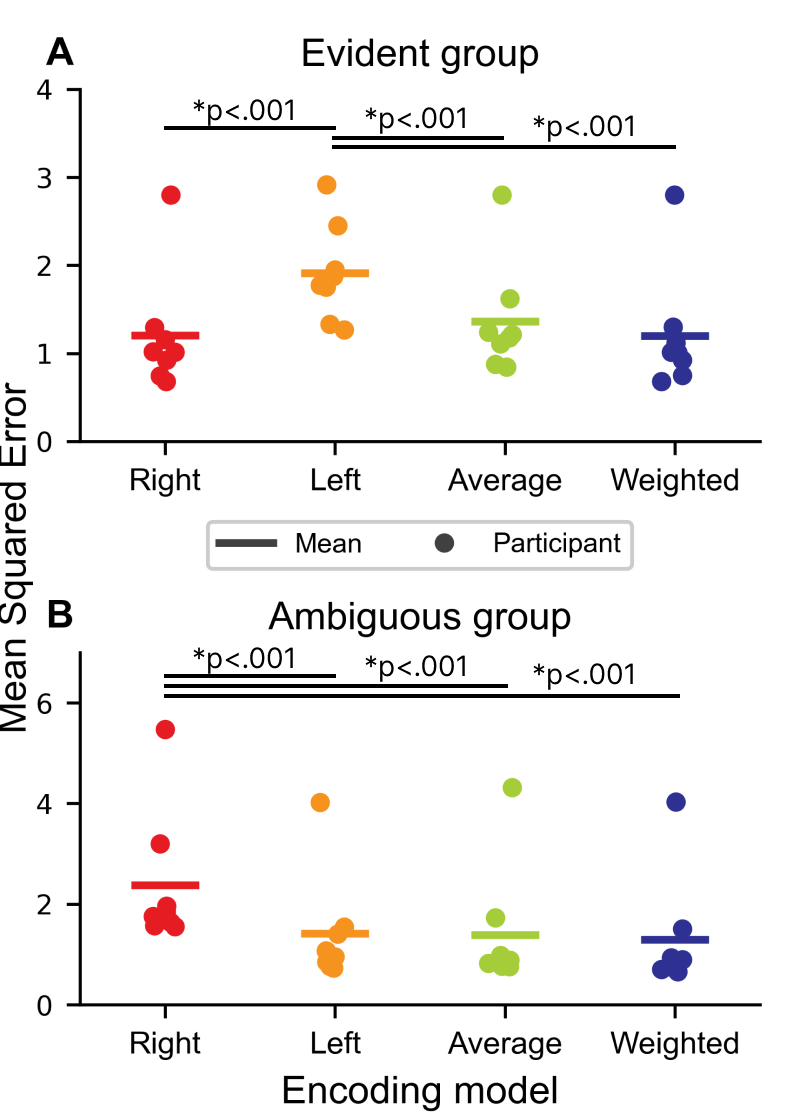

Supplement: S2 Fig — The results show individual data (dots) and the group average (horizontal line) of the MSE during the generalization phase. (A), The evident group showed the lowest MSE for right-hand, average and weighted encoding, with being significantly lower compared to the left-hand encoding. (B), In contrast, the ambiguous group had a lower MSE for left-hand, average and weighted encoding. (TIF) [file pcbi.1011189.s002.tif]
