# Supplementary material for: Learning context shapes bimanual control strategy and generalization of novel dynamics
Source: PLoS Comput Biol. 2023 Dec 8;19(12):e1011189. doi: 10.1371/journal.pcbi.1011189 (PMC10732368; doi:10.1371/journal.pcbi.1011189)
Supplement: Attachment — Submitted filename: Response to the reviewers_PloS_CompBiol.pdf [file pcbi.1011189.s003.pdf]

Response to the reviewers

Content

I. Response to the editor **1**
II. Response to the reviewers **3**
II.I. Response to Reviewer 1 3
II.II. Response to Reviewer 2 5
II.III. Data availability statement 7
III. Changes in figures **8**

I. Response to the editor

As with all papers reviewed by the journal, your manuscript was reviewed by members of the editorial board and by several independent reviewers. The reviewers found that the study was well designed and well conducted, with mostly clear results and conclusions, but did raise some substantial concerns. In particular, the application of computational models is not as thorough as would normally be expected for papers published in PLOS Computational Biology. Given that the application of computational models is central to this journal we have to regard this as a significant weakness of the paper. We would, however, be willing to consider a revised version of the paper if you can address this, along with other weaknesses highlighted by the reviewers.

One suggestion for streamlining the computational part of the paper is that presenting and fitting a multitude of closely related models (Equations 3-7) is overly complicated when in fact all the models considered are special cases of a general model in which the predicted force is a linear combination of left and right hand velocities. It may be more straightforward to fit this two-parameter model (which is basically equivalent to the weight model) and interpret the estimated parameters, rather than comparing 5 distinct models, some of which are quite arbitrarily chosen (e.g. the average and subtract models). A clearer evaluation and presentation of how well this model accounts for behavior across the different generalization conditions (ideally on conditions outside those used to fit the model) would also help improve the modeling results.

I wanted to add a few other comments on top of those of the reviewers. First, before Jackson and Miall, there was also a paper from Bays and Wolpert (J Neurosci, 2006) which considered a similar paradigm and ought to be cited. You might also look at Krakauer et al., PLOS Biology, which examined generalization of shoulder/wrist movements, rather than left hand/ right hand, but contains a similar idea. Third, the attribution of the force to either the left or right hand is very reminiscent of compound conditioning paradigms in which e.g. a light and a tone are presented together, then one is subsequently removed. See e.g. Gershman 2015, PLOS Computational Biology for a review of models of this type of task,

which might be pertinent. A model along these lines was also included in Krakauer et al., 2006, and somewhat related ideas are discussed in Avraham eLife, 2022. You may find that these are interesting parallels to at least discuss.

In light of the reviews (below this email), we would like to invite the resubmission of a significantly-revised version that takes into account the reviewers' comments. In particular, in this revision, we would expect the computational parts of the paper to be substantially improved, although all other reviewer concerns should also be addressed. We cannot make any decision about publication until we have seen the revised manuscript and your response to the reviewers' comments. Your revised manuscript is also likely to be sent to reviewers for further evaluation.

We thank the editor very much for your additional helpful suggestions to improve the paper. In particular we think that your suggestions can help bridge the different suggestions from each reviewer about how to support our models. We have extensively revised the model fitting as suggested. While we still present the additional models (as requested previously), we now focus more on the weighted model and show that it best fits the experimental data. In addition we have revised the text including your additional suggestions.

In particular we have :

- Included a model comparison for the trained condition and evaluated the goodness of fit via R^2 (ll. 185ff and new figure 5)
- Included a model comparison for the bimanual, generalized conditions and evaluated the goodness of fit (ll. 326ff and new figure 7)
- We re-created the figure for predictions and switched from peak force to force compensation (see figure 6, accompanying text ll 235ff). Moreover, we clearly state that the parameters are only fitted to trained conditions of trials in the generalization phase (ll. 200), and that predictions are only generated for generalized conditions.
- We removed the subtract model from our analysis. However, we kept the average model as it might represent a naive state following our argumentation.
- We added the Bays and Wolpert paper to the introduction (ll. 29ff)
- Compound conditioning paradigms: we thank the editor for his feedback and the idea of including the compound conditioning paradigms discussed in Gershman 2015, Avraham 2022 and Krakauer 2006. We added a paragraph in the discussion evaluating the associative learning theory (ll. 448).

II. Response to the reviewers

II.I. Response to Reviewer 1

The authors presented clear evidence that during bimanual movement, one arm can refer to the other arm's motion state when learning to resist the mechanical load induced by the other arm. The authors also showed that this attribution to the arms' motion states is dependent on the degree how the motion states of two arms are correlated; If the motion states of the arms are de-correlated, one can learn the correct association between the load to the left arm and motion of the right arm. On the other hand, when the states are perfectly

correlated (i.e., both arms were moving in the same way), the load induced by the right arm motion was partially attributed to the motion of the left arm. Although this finding is somewhat expected, a more interesting finding is that despite such a difference in attribution, the overall transfer of bimanual learning to the unimanual movement was less affected by the training context. The results presented are informative for the motor learning community. Overall, the manuscript is well-written, and the results are clear. The authors did a good job addressing the concerns raised in the previous round. I only have several minor points that can be easily addressed.

We thank the reviewer for their positive and helpful comments about our manuscript. We have revised the manuscript according to your comments.

Minor Comments:
Line#157-158 and line# 266-267: Given the results of unimanual conditions (Fig. 7E), it looks ambiguous group shows modulation by the speed in the early exposure phase (Fig. 4G). Though this may be simply due to the use of different ANOVA, a brief mention of this could be helpful.

We thank the reviewer for the comment. While the results are not significant in the ANOVA, this trend is indeed interesting and might also be one explanation for the unimanual results. We added a short section on this interesting behavior in ll. 518ff.

Line# 176-260: ANOVA results are missing. Referring to appropriate figure panels (e.g., Fig. 5A, B) would largely help readers to understand the results of bimanual conditions. Figures 5B and 5D seem not referred to in the Results section.

We agree with the reviewer. We changed Figure 5 from peak forces to force compensation as the dependent variable, as we think it increases the readability of the results (now Figure 6). We added the rmANOVA to the text (ll. 238ff) and included statements about the MSE ANOVA (Fig 6 C and D) as well (ll.268ff).

Line#192-193: B is omitted here.

We agree with the reviewer, however, this part has been changed (see ll. 194ff in the mark-up version).

Figure 5C and D: If I correctly understood, the models were fitted to training condition data and then tested on the data for generalization conditions. It is unclear whether the MSEs presented here include that for the training data. In the context of cross-validation, the MSEs for the fitting (i.e., MSEs for training condition data) should be excluded from the evaluation.

We agree. We have now extensively revised the analysis and figures. The BIC and MSEs are clearly separated for the training conditions only (Figure 5 for the BIC) and the generalization conditions only (Figure 6 for the MSE and Figure 7 for the BIC) in the new

figures. We confirm that the MSEs for the fitting (the training condition) are not included in the evaluation on the generalization conditions. We now explicitly mention this in the figure caption.

Figure 5D: Missing x-labels. The presentation of Figure 5 could be improved. For instance, the data for bimanual and unimanual conditions could go into separate figures, as the current version is too visually crowded. Also, using lines in Fig. 5C and D is not very informative. Simply using dots arranged horizontally can improve visibility in the same space. Panel D can be only for unimanual conditions and probably go to separate figures with unimanual peak force data (from panels A and B).

We agree with the reviewer. We have extensively revised the figures to make them more readable in addition to presenting the material differently. Figure 5D has now been removed. We also agree that the unimanual conditions were not required in this figure and have separated the unimanual conditions within the manuscript.

As there are separate panels for the two groups, actual force values do not need different colors. Currently, it is visually hard to detect lines in similar colors (e.g., red/orange lines over brown shade). Gray shade could be nice for getting good contrast with all the colors used.

We thank the reviewer for this point. While we have kept the two different colors for the two experimental groups, we have extensively revised the figures to try to make the specific results clearer to the readers.

Line#609: Referring to wrong Eq. # (should be seven instead of 5).

We thank the reviewer. This has been changed to equation 6.

Line#594-619 (regression analysis): Were the alpha values also constrained to [0,1]? If so, it might be unfair to the subtraction coding model (perhaps the presence of B requires the constraint on alpha, but the B in the models looks redundant). Please clarify.
Please also specify how unimanual conditions were simulated (by simply setting x_dot_right to 0?).

We thank the reviewer. We have revised the analysis and answer based on the current version of the manuscript. The values of alpha and omega were not constrained. We removed the subtract model from the analysis, as the results were not meaningful and did not provide any additional insight. B reflects the force field strength of 0.13N. The unimanual conditions are simulated by using the different model equations, while using the generated velocity vector of the left arm and setting the velocity vector of the right arm to 0. These are all now added to the manuscript.

II.II. Response to Reviewer 2

Reviewer #2: In this work, the authors probe the influence of speed or movement length as a factor in adopting a bimanual control strategy. They perform an experiment wherein the

speed of right hand movement determined the lateral force applied to the left hand. Two experimental groups were employed, both utilizing the velocity of right hand movement to determine the left-hand forces while the right hand always moved in a null field. In the “ambiguous” group, both hands moved at identical speeds and distances, creating ambiguity for the sensorimotor system in utilizing the sensory state of either hand to plan subsequent movements. In contrast, for the “evident” group, there was a distinction in speed between the hands, making it evident to the sensorimotor system that right hand speed was more relevant for predicting the force-field applied to the left hand. Generalization trials, incorporating interspersed error clamp catch trials, were used to assess the encoding scheme utilized in forming the bimanual memory. The authors report that the evident group employed a right hand encoding scheme, whereas the ambiguous group adopted an average or weighted average encoding scheme for bimanual memory formation.

We thank the reviewer for their positive and helpful comments about our manuscript. We have extensively revised the manuscript according to your comments.

1. How the motor system adapts to perturbations applied during bimanual movements is not well understood, and the literature on this topic is also limited. The study therefore addresses an important issue, but the findings are hardly profound or ground-breaking. The larger conclusion that training conditions strongly influence the encoding of a motor memory, and hence also shape subsequent generalization, is well-known from work in unimanual movements. The current study extends this to bimanual action conditions, but does not break new ground in terms of deepening our understanding about the influence of training conditions.

We thank the reviewer for bringing up this point. One of the novel additions of our manuscript is that movement of the non-adapting limb isn’t only acting as a contextual cue, but instead the motor memory is encoded based on the actual kinematics of the movement. For example, previously in the key papers by Daichi Nozaki or Ian Howard, the motion of one arm cues a specific motor memory that is selected and used for the other arm. However, in our task it is actually the velocity of the other arm which affects the overall force generalization of the motor output. That is we are (at least partially) encoding the information of the right arm to predict actions on the left arm. Moreover, we show that this encoding is gradually developed as the participants experience the environment. Early in the adaptation process, we find evidence that the initial motor memory may represent a unimanual movement and that the weighting of the bimanual component is then developed over training. We do not know of any manuscripts investigating unimanual movements that can show such development of bimanual motor memories. We have added some additional sentences to the discussion outlining this contribution.

2. I also think that the authors have over-interpreted their results in favor of what has been ultimately reported. Specifically, it is not at all clear to me why the average encoding isn’t the best predictor of how people adapt left arm dynamics in either group. The authors do consider this possibility for the ambiguous group (and this makes sense), but I am not convinced that the right hand encoding does better than the average encoding for the evident group. All the plots of figure 5 and the statistics also point to average encoding doing

better even for the evident group. The authors are requested to present key metrics that will convince a reader that

We thank the reviewer for this point. We actually argue that weighted encoding, rather than average encoding, best describes both experiments. However, we now provide additional support for this with BIC and MSE measures across both experiments and for both trained conditions (used to fit model parameters) and generalization conditions (not used to fit model parameters). Moreover we do this for individual participants as well as the whole group. While the MSE (see Figure 6) is only slightly higher for the average model compared to the weighted model, the BIC for both the trained and generalized conditions show a clear picture: the right hand and weighted encoding outperform the average model in all participants, in both groups. We hope to convince the reader with the novel, provided evidence.

3. Control groups with force fields applied to the left arm under unimanual conditions are sorely missing from the study. While it may be obvious that predictive force development will be associated only with left arm kinematics in this case, it is important to know how the left arm contribution during bimanual actions, particularly in the ambiguous case, emerges/changes relative to the unimanual condition.

We thank the reviewer for this point. In the unimanual case, we can only assume pure left hand encoding. Only after switching from a unimanual to bimanual setting (e.g. during generalization), we could assess the transferred encoding weights. However, the primary purpose of the present study was to investigate whether participants could learn the actual mapping of right hand kinematics to the left arm. Compared to other authors, the movement of the right hand did not only serve as a cue, but changed the applied forces on the left arm. Moreover, Nozaki (2006) and Yokoi et al (2011) showed that the switch between unimanual and bimanual only leads to partial transfer, which has been confirmed by the present study as well. Consequently, we will probably see similar effects and a naive state employing an average encoding scheme early in adaptation.

4. The modeling Results are very under-reported. The authors have made somewhat sweeping statements about one encoding model being better or worse than another. However, no statistical comparison across different models is available. The authors must provide a solid comparison across models (using AIC, etc.) so that one can make more informed and reliable conclusions about which encoding method works best in the two cases.

We agree. This is an important point that was missing from our manuscript. We have added additional support based on BIC and MSE across the conditions, both for the conditions that we fit the model on (Figure 5), and for the generalization conditions which are not used to fit the model parameters (Figure 7). Moreover, we added the R^2 values to each of the latter figures as a measure of goodness of fit. Figure 6 shows the generated force compensation as well as the predictions for each model in novel, generalized conditions. To quantify the amount of adherence to each model, we calculated the MSE for model (the same analysis was done for the peak forces, see Supplementary Material S2 Appendix).

5. This study has a very limited sample size – only 8 subjects per group. Our field of work has gained some disrepute due to small sample sizes and most labs are striving to do better. The authors are therefore requested to provide a justification for why such a small sample size is good enough for making sound conclusions.

We thank the reviewer for this comment. First, it is important to note that each participant performed the experiment for between 5-6 hours, which is different from many experiments, for example in visuomotor rotation, that last only 30 minutes. This allows us to accurately measure the response of each participant. Secondly, the measurements for each participant were consistent and showed clear results. Thirdly, and most importantly, we now show the BIC and MSE values not only across the conditions but also for each individual participant. Almost all participants show the same results and trends as the overall results, so our findings don't rely only on some small statistical test on the mean results across participants. We argue that this is more robust than adding a few more participants.

Minor:

1. Is the right arm encoding line missing from the bff condition in Figure 5B? I cant seem to find the red line.

Thank-you. The red line was hidden by the other lines. We have now changed the figure to distribute them along the x-axis. (see Supplementary Material S1 Appendix)

2. line 609: "Initial ω in (5) was set to 0.5". There is no ω in eq. 5.

The text should have referred to equation 6 (please note, that the equations were changed for the new version). This has now been corrected.

II.III. Data availability statement

Reviewer #1: **No:** The authors state the data will be made available on Figshare before publication.

Reviewer #2: **No:** I could not find a statement on data and code availability.

The raw data underlying all figures is published in Figshare under the following link: https://doi.org/10.6084/m9.figshare.22895378.v1

III. Changes in figures

- Fig 5 was adapted and moved to the supplementary material as "S1 Appendix".
- Fig 5 was replaced by a new figure displaying the force compensation instead of the peak forces to account for both, the required forces and the generated forces and depict them in a single value (now Fig 6)

- Fig 5 (new) (BIC model comparison for trained conditions) was added
- Fig 7 (new) (BIC model comparison for generalized conditions) was added
- S2 Appendix Fig (Mean Squared error for generalized conditions of force profiles) was added
- Fig 9 (Unimanual force profiles) was changed to show the different predictions
